# Study of Coagulation Parameters in Gastrointestinal Malignancies

**DOI:** 10.7759/cureus.72162

**Published:** 2024-10-22

**Authors:** Gauri Patil, Sujata R Kanetkar

**Affiliations:** 1 Department of Pathology, Krishna Institute of Medical Sciences, Krishna Vishwa Vidyapeeth (Deemed to be University), Karad, IND

**Keywords:** aptt, coagulation, d-dimer, gastrointestinal, malignancies, prothrombin, thrombosis

## Abstract

Introduction

Gastrointestinal (GI) malignancies represent a diverse group of cancers affecting various parts of the digestive system. These malignancies encompass an important burden of cancer incidence and mortality globally, contributing to substantial morbidity and mortality worldwide. Studying the coagulation parameters of patients having GI malignancies is crucial for several reasons. It allows to identify the patients at an increased risk of thrombotic complications, enabling clinicians to implement appropriate prophylactic measures, such as anticoagulant therapy or mechanical thromboprophylaxis.

Aim

To study coagulation parameters in patients diagnosed with GI malignancies.

Materials and methods

The present study is a two-year prospective observational study, carried out in the Department of Pathology in our tertiary care institute from July 2022 to June 2024 to investigate the coagulation profile in patients diagnosed with GI malignancies. A total of 86 cases were studied.

Results

A significant increase in the mean values of coagulation parameters was noted with an increase in the grade of malignancy.

Conclusion

Early examination for the presence of coagulation abnormalities can help to prevent morbidity and mortality and other bleeding diathesis in GI malignancies as alterations in the coagulation pathway can lead to lethal complications.

## Introduction

Gastrointestinal (GI) malignancies encompass a wide range of cancers that impact different parts of the digestive system, including the esophagus, stomach, small intestine, colon, rectum, and anus. These cancers significantly contribute to global cancer incidence and mortality, leading to considerable rates of morbidity and mortality worldwide. A thorough understanding of the pathophysiology, prognosis, and treatment options for GI malignancies is essential for effective patient care and better health outcomes.

The relationship between coagulation and cancer has been recognized for decades. Research indicates that cancer patients have a heightened risk of developing thrombotic complications. This dysregulation of coagulation can arise from various factors, including tumor-derived procoagulant substances, inflammation, endothelial dysfunction, and imbalances between procoagulant and anticoagulant mechanisms. Consequently, grasping the complex interaction between cancer and coagulation is crucial for formulating strategies to prevent and manage thrombotic events in cancer patients [1].

Nevertheless, studying coagulation in cancer patients, particularly those with GI malignancies, presents several challenges. Additionally, determining the best approach to manage thrombotic complications in these patients is an area of ongoing investigation, highlighting the need for prospective studies to establish evidence-based guidelines [2].

## Materials and methods

Study design

This two-year prospective observational study was conducted in the Pathology Department of our tertiary care institution from July 2022 to June 2024. The aim was to examine the coagulation profile in patients diagnosed with GI malignancies.

Sample size

The study included 86 cases of GI malignancies. The sample size was calculated using the formula n = 4pq/L^2^,where n is the number of cases.

Inclusion and exclusion criteria

We included patients who were clinically suspected, radiologically evaluated and histopathologically diagnosed with GI malignancies. Patients with other malignancies, with preexisting coagulation disorders (such as thrombosis or hemophilia), on anticoagulant therapy (like warfarin or heparin) prior to cancer diagnosis, or with significant comorbidities that could affect coagulation parameters were excluded.

Methodology

Blood samples were collected from the patients in the general and onco-surgery departments who had clinical or radiological suspicions of GI malignancies and were subsequently diagnosed through histopathological examination. We assessed parameters such as platelet count, prothrombin time (PT), activated partial thromboplastin time (aPTT), and D-dimer levels. Venous blood samples were obtained using trisodium citrate and ethylenediaminetetraacetic acid vacutainers. A Nihon Kohden five-part analyzer measured platelet counts, aligning with peripheral smear findings. PT and aPTT were determined using the Satellite Max machine (Diagnostica Stago S.A.S., Asnières-sur-Seine, France) while D-dimer levels, indicative of fibrinolysis and thrombus formation, were also quantified using the same device.

Statistical analysis

Data were compiled in MS Excel and analyzed using SPSS 19.0 (IBM Corp, Armonk, NY). The appropriate ANOVA (analysis of variance) test was applied to assess the variables, and a p-value of less than 0.05 was considered statistically significant.

## Results

A total of 86 cases of GI tract malignancies were examined in this study. Patient ages ranged from 30 to 90 years, with the highest number of cases found in the 61-70 age group. A male predominance was noted among the cases. The most common presenting symptoms were abdominal pain and weight loss, reported in 35 cases (40.69%). The GI malignancies identified included those in the colon, esophagus, stomach, gastroesophageal junction, rectum, and anal canal, with the colon being the most frequently affected site. Adenocarcinoma was the predominant histological type, accounting for 72 cases (83.72%) out of 86. This category included well-differentiated to poorly differentiated adenocarcinomas based on histological grading at diagnosis. The second most common type was squamous cell carcinoma (SCC), which comprised 14 cases (16.27%) (Table 1). The platelet counts in adenocarcinoma patients ranged from less than 0.15 million to over 0.45 million, with 43 cases (50%) having normal platelet counts. Twenty-one cases (29.16%) exhibited thrombocytopenia, while eight cases (11.11%) showed thrombocytosis. Among patients with SCC, 10 cases (71.42%) had normal platelet counts, one case (7.14%) had thrombocytopenia, and three cases (3.48%) had thrombocytosis.

**Table 1:** Distribution of cases of gastrointestinal malignancies according to site and histological subtype

Site	Adenocarcinoma	Squamous cell carcinoma
Esophagus	4	14
Stomach	20	-
Gastroesophageal junction	2	-
Colon	31	-
Rectum	10	-
Anal canal	5	-

Coagulation parameters, including PT, aPTT, and D-dimer, were compared across all grades of malignancies. The mean PT values were 16.48 ± 0.82 seconds for well-differentiated adenocarcinoma, 18.29 ± 0.96 seconds for moderately differentiated adenocarcinoma, and 20.39 ± 1.33 seconds for poorly differentiated adenocarcinoma. The mean aPTT values were 39.22 ± 0.94 seconds for well-differentiated, 42.61 ± 1.34 seconds for moderately differentiated, and 45.25 ± 1.44 seconds for poorly differentiated adenocarcinoma. The mean D-dimer levels were 0.59 ± 0.07 for well-differentiated, 1.29 ± 0.16 for moderately differentiated, and 2.68 ± 0.83 for poorly differentiated adenocarcinoma (Table 2).

**Table 2:** Distribution of mean values of adenocarcinoma cases with relation to coagulation parameters PT, prothrombin time; aPTT, activated partial thromboplastin time; p, probability; SD, standard deviation.

Coagulation parameters	Well differentiated		Moderately differentiated		Poorly differentiated		p-Value
	Mean	SD	Mean	SD	Mean	SD	
PT	16.48	0.82	18.29	0.96	20.39	1.33	<0.0001
aPTT	39.22	0.94	42.61	1.34	45.25	1.44	<0.0001
D-Dimer	0.59	0.07	1.29	0.16	2.68	0.83	<0.0001

Reference range: PT: 12.50-16.50 seconds, aPTT: 27-38 seconds, D-dimer: 0.1-0.5 µg/ml, platelet: 1.5-4.5 lacs.

Coagulation parameters, including PT, aPTT, and D-dimer levels, were compared across all grades of SCC. The mean PT was 15.66 ± 1.15 seconds for well-differentiated SCC, 17.74 ± 1.28 seconds for moderately differentiated SCC, and 19.92 ± 0.83 seconds for poorly differentiated SCC. The mean aPTT values were 37.66 ± 2.08 seconds in well-differentiated SCC, 41.57 ± 2.22 seconds in moderately differentiated SCC, and 44.75 ± 2.50 seconds in poorly differentiated SCC. Additionally, the mean D-dimer levels were 0.93 ± 0.28 in well-differentiated SCC, 1.20 ± 0.08 in moderately differentiated SCC, and 1.95 ± 0.30 in poorly differentiated SCC (Table 3).

**Table 3:** Distribution of squamous cell carcinomas with relation to coagulation parameters PT, prothrombin time; aPTT, activated partial thromboplastin time; p, probability; SD, standard deviation.

Coagulation parameters	Well differentiated		Moderately differentiated		Poorly differentiated		p-Value
	Mean	SD	Mean	SD	Mean	SD	
PT	15.66	1.15	17.74	1.28	19.92	0.83	<0.0018
aPTT	37.66	2.08	41.57	2.22	44.75	2.50	<0.0064
D-Dimer	0.93	0.28	1.2	0.08	1.95	0.30	<0.0001

## Discussion

The age range of patients in this study was 30-90 years, with the highest number of cases observed in the 61-70 age group (33.73%), followed by the 51-60 age group (27.91%). The youngest patient was 35 years old, while the oldest was 90 years old. These findings align with those of Bhubaneshwar Saikia et al. [3]. Among the 86 cases studied, there was a male predominance, with 50 males (58.14%) and 36 females (41.86%). This distribution is consistent with the findings of Rema Nair Sarkar et al. [4] and Shakuntala et al. [5].

In this study, the most common presenting symptoms were abdominal pain and weight loss, seen in 35 cases (40.69%). This is similar to the findings of Habeebu et al. [6], where abdominal pain was the primary complaint. The GI malignancies observed included the colon, esophagus, stomach, gastroesophageal junction, rectum, and anal canal, with the colon being the most frequently affected site (31 cases, or 36.04%), followed by the stomach (20 cases, or 23.26%). These results corroborate the studies by Rema Nair Sarkar et al. [4] and Habeebu et al. [6].

Platelet counts in adenocarcinoma patients ranged from less than 0.15 million to more than 0.45 million. Forty-three cases (50%) had normal platelet counts, 21 cases (29.16%) had thrombocytopenia, and only eight cases (11.11%) exhibited thrombocytosis. In SCC patients, 10 cases (71.42%) had normal platelet counts, one case (7.14%) had thrombocytopenia, and three cases (21.42%) had thrombocytosis. These findings are in agreement with the study by Bhubaneswar Saikia et al. [3] and Fitalew Tadele Admasu et al. [7].

Coagulation parameters, including PT, aPTT, and D-dimer levels, were compared across all grades of adenocarcinoma. The mean PT was 16.48 ± 0.82 seconds for well-differentiated adenocarcinoma, 18.29 ± 0.96 seconds for moderately differentiated adenocarcinoma, and 20.39 ± 1.33 seconds for poorly differentiated adenocarcinoma. The mean aPTT values were 39.22 ± 0.94 seconds for well-differentiated, 42.61 ± 1.34 seconds for moderately differentiated, and 45.25 ± 1.44 seconds for poorly differentiated adenocarcinoma. Mean D-dimer levels were 0.59 ± 0.07 for well-differentiated, 1.29 ± 0.16 for moderately differentiated, and 2.68 ± 0.83 for poorly differentiated adenocarcinoma. A significant increase in the mean values of coagulation parameters was observed with higher grades of malignancy, consistent with the studies by Fitalew Tadele Admasu et al. [7], Kiran Shaikh et al. [8], Linzhang et al. [9], and Mashio Nakamura et al. [10].

Limitations

This study was limited by a small sample size, which was influenced by the high cost of reagents, imaging studies, and patient compliance. Additionally, the impact of treatment on these parameters was not examined.

Strengths

Clinical Relevance

The research addresses the critical link between cancer and coagulation disorders, which is essential for the prognosis and treatment of GI malignancies. Understanding this relationship can guide better management of thrombotic complications, a common issue in cancer patients.

Prospective Design

The study was conducted as a two-year prospective observational study. This approach strengthens the findings by reducing recall bias and allowing the researchers to track changes over time.

Comprehensive Data Collection

It covers various aspects of coagulation, including platelet count, PT, aPTT, and D-dimer levels. This extensive data collection allows for a holistic understanding of coagulation abnormalities in GI cancer patients. *Sample Size and Diversity*

With 86 cases, the study seems to have a reasonable sample size for assessing coagulation profiles in GI cancers, providing a good basis for statistical analysis. The sample includes a range of GI malignancies from different sites, ensuring diverse data.

Detailed Histopathological Analysis

The study differentiates between various histological types and grades of malignancies (adenocarcinoma, squamous cell carcinoma), correlating them with coagulation parameters. This enables specific insights into how tumor characteristics affect coagulation.

## Conclusions

Several coagulation profile parameters are elevated in GI malignancies. The results of these tests seem to be affected by the severity of clinical findings, as well as the histological types and grades of the cancers. Adenocarcinoma exhibited more significant alterations in the coagulation profile compared to SCC. Higher grades of malignancies were positively associated with abnormal coagulation results. Early detection of coagulation abnormalities can help prevent morbidity, mortality, and bleeding diatheses in GI malignancies, as changes in the coagulation pathway can lead to serious complications.

However, further studies are required, with a larger patient size and a detailed follow-up for recording the mortality related to coagulation pathway dysregulation. 

## References

[1] Elyamany G, Alzahrani AM, Bukhary E (2014). Cancer-associated thrombosis: An overview. Clin Med Insights Oncol.

[2] Frere C (2021). Burden of venous thromboembolism in patients with pancreatic cancer. World J Gastroenterol.

[3] Saikia B, Khandelia R (2018). Study of haematological and coagulation profile in patients with gastrointestinal malignancies. Glob J Res Anal.

[4] Sarkar RN, Kartheek BVS, Satish SK, Bhagyalakshmi A (2017). Study of gastrointestinal cancers in a tertiary care hospital. J Evid Based Med Healthc.

[5] Shakuntala TS, Krishnan SK, Das P (2022). Descriptive epidemiology of gastrointestinal cancers: Results from National Cancer Registry Programme, India. Asian Pac J Cancer Prev.

[6] Habeebu MY, Salako O, Okediji PT, Mabadeje B, Awofeso OM, Ajekigbe AT, Abdulkareem FB (2017). The distribution, histologic profile and clinical presentation of gastrointestinal malignancies in Lagos, Nigeria. J West Afr Coll Surg.

[7] Admasu FT, Dejenie TA, Ayehu GW (2023). Evaluation of thromboembolic event, basic coagulation parameters, and associated factors in patients with colorectal cancer: A multicenter study. Front Oncol.

[8] Shaikh K, Nizamani GS, Nizamani YM, Nizamani N, Fahim A, Nadeem F (2020). Altered coagulation pattern in different histological grades of squamous cell carcinoma. J Islamabad Med Dental Coll.

[9] Zhang L, Ye J, Luo Q, Kuang M, Mao M, Dai S, Wang X (2020). Prediction of poor outcomes in patients with colorectal cancer: Elevated preoperative prothrombin time (PT) and activated partial thromboplastin time (APTT). Cancer Manag Res.

[10] Nakamura M, Sakon M, Sasako M (2024). Association of D-dimer level with thrombotic events, bleeding, and mortality in Japanese patients with solid tumors: A Cancer-VTE Registry subanalysis. Int J Clin Oncol.

